# Truncating SOX9 Alterations Are Heterozygous Null Alleles in Genome-Stable Colorectal Cancer

**DOI:** 10.1016/j.gastha.2022.04.011

**Published:** 2022-05-02

**Authors:** G. N. DURONIO, X. LIANG, P. HEBBAR, M. ISLAM, S. SPISAK, N. S. SETHI

**Affiliations:** 1 Division of Molecular and Cellular Oncology, Department of Medical Oncology, Dana-Farber Cancer Institute, Boston, Massachusetts; 2 Department of Gastroenterology, The First Affiliated Hospital of Chongqing Medical University, Chongqing, China; 3 Center for Functional Cancer Epigenetics, Dana-Farber Cancer Institute, Boston, Massachusetts; 4 Cancer Program, Broad Institute of Massachusetts Institute of Technology (MIT) and Harvard University, Cambridge, Massachusetts; 5 Department of Medical Oncology, Gastrointestinal Cancer Center, Dana-Farber Cancer Institute, Boston, Massachusetts; 6 Department of Medicine, Brigham and Women’s Hospital and Harvard Medical School, Boston, Massachusetts

Through an integrative analysis leveraging patient-derived molecular information, we recently defined the genome-stable subtype of colorectal cancer (CRC), a previously unrecognized subgroup that lacks significant aneuploidy and elevated mutational density.^1^ A striking molecular feature of this new class is the presence of highly recurrent mutations in the developmental transcription factor and wingless-related integration site (WNT) pathway target *SOX9*.^2^ However, the functional significance of these alterations in CRC remains poorly understood. Prior studies hypothesized a gain-of-function role for the mutant SOX9 based on genomic analyses of human CRC cases.^3,4^ However, to date, a direct functional analysis of the role of truncated SOX9 proteins has yet to be performed. In this *research letter*, we annotate SOX9 mutations in CRC, describe their transcriptional and epigenomic consequences, and postulate as to why they are selected for in genome-stable CRC.

In CRC, *SOX9* alterations are predominantly nonsense/frameshift mutations that preferentially cluster in 3 functional domains found within the c-terminal half of the gene (Figure 1A and B). We confirmed that truncated forms of SOX9 are expressed in a subset of CRC cell lines harboring endogenous mutations (Figure A1A and B), often at higher levels than endogenous wildtype (WT) SOX9. Genomic analyses of human CRC from The Cancer Genome Atlas and Cancer Cell Line Encyclopedia indicated that the majority of *SOX9* mutations are heterozygous, preserving a WT copy of the gene (Figure 1C). This result raised a few possibilities as to the function of mutant truncated SOX9 proteins: (1) They carry gain-of-function properties by regulating a distinct transcriptional program, (2) they exert dominant-negative activity by inhibiting the WT SOX9 function, or (3) they behave as a null allele leading to a heterozygous state.

To investigate the transcriptional and epigenomic consequence of mutant SOX9 as well as distinguish between these possibilities, we pursued a comprehensive genome-wide molecular analysis of mutant and WT SOX9. We conditionally overexpressed 4 SOX9 protein constructs with N-terminal V5 protein tags in HT-115 CRC cells. These included 1 WT construct and 3 mutant SOX9 alleles with sequential loss of its c-terminal domains, representing the spectrum of mutations observed in patients (Figure 2A and B). Truncated SOX9 mutants were expressed at higher levels than WT SOX9, which may reflect greater tolerance to elevated expression of truncated variants. The V5 protein tag ensured the specific assessment of the conditionally overexpressed WT and mutant SOX9 without engaging endogenous SOX9 expressed in the cells (Figure 2C). Genome-wide binding of mutant and WT SOX9 was determined using chromatin-immunoprecipitation followed by DNA sequencing (ChIP-seq) using an anti-V5 antibody. Mutant and WT SOX9 were bound to identical locations throughout the genome (~1750 sites, Figure 2D); the motif analysis of these sites showed greatest enrichment for the native SOX9 binding sequence as determined by SeqPos (*P* = 1 × 10^−336^). Histone H3 lysine 27 acetylation (H3K27Ac) ChIP-seq, which marks active enhancers and promoters participating in transcriptional regulation, was also performed on these cell lines. All V5- and H3K27Ac-ChIP sequencing analyses were performed on 2 high-quality replicates in each experimental condition. Most of the loci identified contain a moderate, baseline level of H3K27Ac due to endogenous WT SOX9 activity, as shown by green fluorescent protein expressing control cells. However, enrichment analysis indicated that WT but not mutant SOX9 binding increased H3K27Ac and thus transcriptional activity when compared to the green fluorescent protein control (Figure 2E). Integration of V5-CHIP-seq data and messenger RNA expression profiles from RNA-sequencing using the binding and expression target analysis pipeline endorsed that only WT SOX9 is functioning as a transcriptional activator, whereas mutant SOX9 variants appear to lack transcriptional activity (Figure 2F). Global H3K27Ac and RNA-sequencing data did not show evidence of mutant SOX9-specific transcriptional activity, suggesting that a gain-of-function behavior is unlikely.

HT-115 CRC cells endogenously express WT SOX9, which enables the evaluation of whether overexpression of mutant SOX9 variants interferes with their transcriptional activity through a dominant-negative effect. Despite binding to the exact same locations, mutant SOX9 did not exert dominant-negative activity as the genes upregulated by WT SOX9 were unaffected by all 3 mutant SOX9 variants (Figure 2F; purple line would be significantly above static dotted line if dominant-negative activity was present). These results revealed that *SOX9* mutations lead to expression of truncated SOX9 proteins that are incapable of canonical transcriptional activity, do not show evidence of gain-of-function properties, and do not interfere with the WT SOX9 function through dominant-negative activity. In other words, *SOX9* mutations likely lead to loss of function, overexpression of nonfunctional mutant alleles, and yield hemizygous expression of WT SOX9.

We next asked whether a truncating mutation can be introduced into WT SOX9 CRC cell lines using CRISPR/Cas9. Directing Cas9 to the c-terminus of *SOX9* using sgRNA#9 in 3 CRC cell lines led to protein expression of a truncated variant at the expected size (Figure A2A). Despite the constitutive expression of the CRISPR/Cas9 machinery, WT SOX9 expression was found when analyzing protein from a pooled population in each of the cell lines. By analyzing single-cell clones (Figure A2B and C), it was clear that homozygous *SOX9* alterations were infrequent. Two CRC cell lines did not tolerate homozygous mutations in any surviving clones, and a third, COLO-205, produced a minority of clones containing a homozygous mutation (3/24). These results suggest that, if mutated, SOX9 is preferentially altered in a heterozygous fashion. Notably, these data also raised the possibility that WT SOX9 is required for CRC as homozygous alterations occur infrequently if at all. These findings were followed up in a recently published companion manuscript^5^ focused on the requirement for WT SOX9 in CRC. Given that ~10% of CRCs harbor a heterozygous SOX9 mutation that preserves a WT copy, it opens further areas of investigation into the advantage provided by a haploinsufficient SOX9 state.

Here, we provide evidence that an important downstream mediator of WNT signaling, SOX9, is preferentially mutated in a heterozygous fashion in CRC. We additionally found that SOX9 mutations cluster in the c-terminal half of the gene, consisting mostly of nonsense and frameshift mutations, and lead to overexpression of truncated forms of SOX9 that lack gain-of-function, dominant-negative, or canonical transcriptional activity. Early studies of SOX9 assigned a potential gain of function to these mutations given their predilection for the c-terminus, leading to overexpression of truncated variants in human tumors.^3,4^ Gain-of-function mutations have been characterized in a number of cancer genes including *TP53*; the pathogenic significance, however, is now under debate with recent evidence showing that mutational processes may shape the disproportionate frequency of hotspot mutations^6^ and that the dominant-negative/loss-of-function behavior may predominate.^7^ When evaluating the functional significance of *SOX9* mutations, we set a high threshold for gain-of-function properties, scrutinizing any evidence of novel DNA-binding, noncanonical gene expression changes, or phenotypic advantages. Ultimately, it appears that truncating mutations lead to a nonfunctional allele, which raises new questions about potential advantages of single-copy gene expression in cancer.

*TCF7L2*, which encodes the essential WNT pathway cofactor TCF4, is also recurrently mutated in a heterozygous fashion in CRC (Figure A2D and Table). The prevalence of heterozygous, inactivating mutations in another essential WNT pathway component suggests a functional cancer-specific advantage to this mutational pattern (Table). Losing 1 copy of a pathway regulator could relieve negative feedback on WNT while still maintaining moderate expression of genes that are essential for preventing terminal differentiation of intestinal cells, such as SOX9.^5^ For example, overexpression of WT but not truncated mutant SOX9 blocks WNT signaling as indicated by reporter activity in 293T cells (Figure A2E). Recently, the inhibitory activity of SOX9 on the WNT pathway was demonstrated to be mediated by MAML2.^8^

Another potential advantage of heterozygous mutations is supported by the dose-dependent nature of SOX9 function, which is exemplified in normal intestinal stem cells where high SOX9 expression promotes quiescence.^6^ This would support the hypothesis that a heterozygous mutation attenuates SOX9 expression while retaining the necessary functions as a WNT mediator and intestinal stem cell factor. A third possibility is that heterozygous *SOX9* mutations confer phenotypic plasticity, defined as the ability to change cell states, which is increasingly being recognized for its contributions to cancer initiation, progression, metastasis, and drug resistance.^9^ We speculate that the acquisition of plasticity may underlie selection of heterozygous null *SOX9* mutations in CRC. This “goldilocks” level of SOX9 protein expression may enable the flexibility to achieve different cellular states when facing selective pressures of neoplasia. Even with the pieces of evidence presented in this study, which was limited by reliance on cancer cell lines, better model systems and more systematic evaluation are required to rigorously examine whether heterozygous *SOX9* mutations confer plasticity. These experiments will be critical for determining the mechanism by which heterozygous *SOX9* mutations serve a functional advantage in CRC.

## Supplementary Material

Supplementary Material

## Figures and Tables

**Figure 1.**
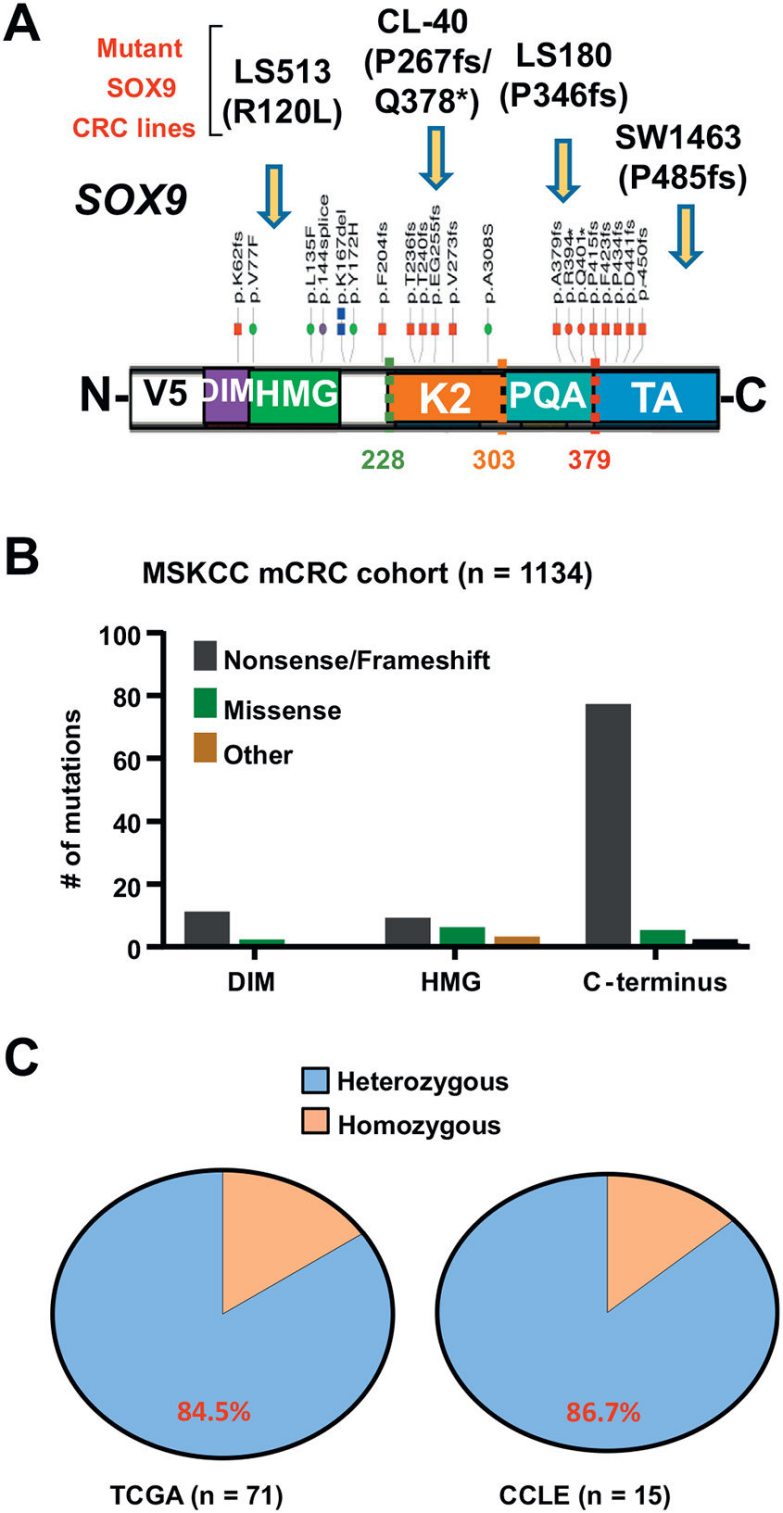
*SOX9* is typically altered by heterozygous mutations that cluster in the c-terminus. (A) Schematic showing functional domains of *SOX9* gene, abbreviated as dimerization domain (DIM), DNA-binding high mobility group (HMG) domain, and transactivation domains (K2 and TA) and a PQA-rich domain (PQA); distribution and types of somatic mutations along gene in TCGA non-microsatellite unstable CRC cohort; CRC cell lines with endogenous *SOX9* mutations (arrows); mutations marked in red indicate truncating mutations; dotted lines correspond to truncated cDNA variants used for overexpression experiments. (B) Quantification of somatic mutations that occur in indicated *SOX9* gene domains in Memorial Sloan Kettering Cancer Center metastatic CRC cohort (n = 1134). (C) Percentages represent frequency of *SOX9* mutations in *SOX9*-altered CRC from TCGA and CCLE cell lines. CCLE, Cancer Cell Line Encyclopedia; CRC, colorectal cancer; TCGA, The Cancer Genome Atlas.

**Figure 2.**
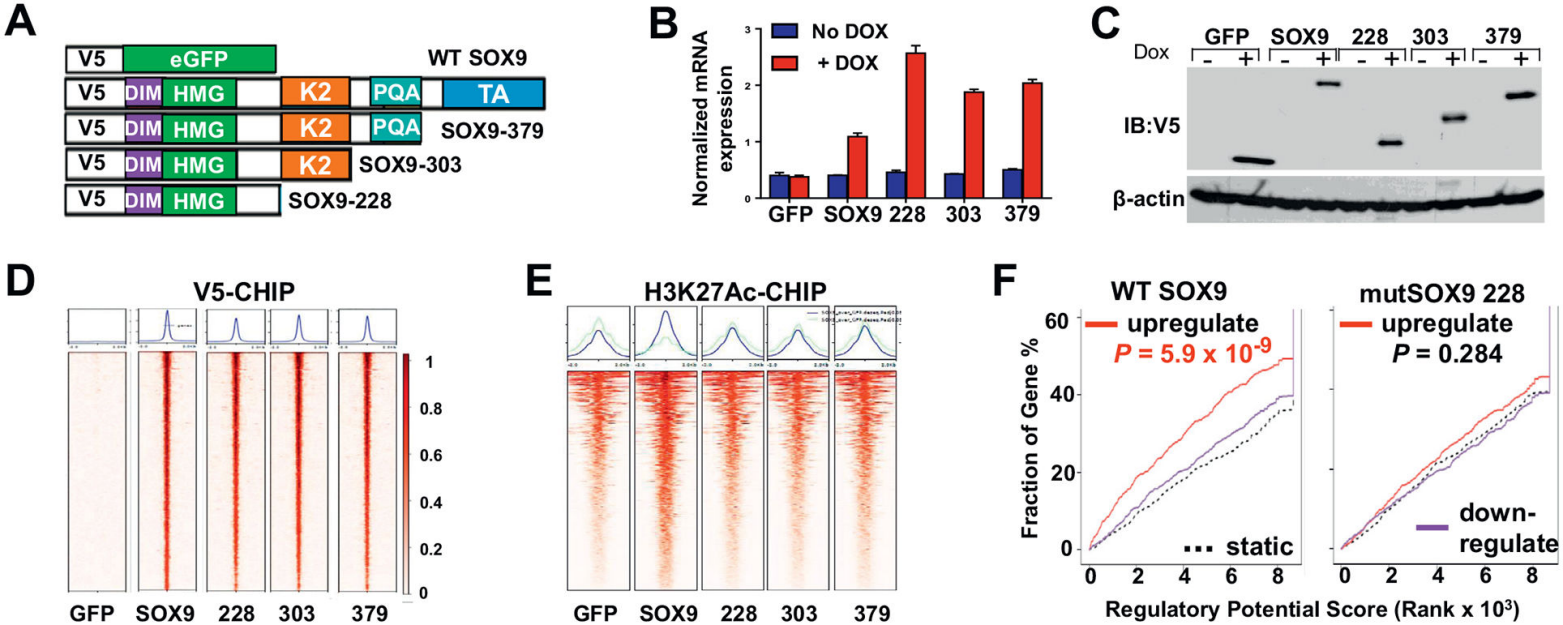
*SOX9* mutations lead to expression of truncated variants without canonical or dominant-negative transcriptional activity. (A) Schematic indicating construction of WT and mutant SOX9 experimental system. (B) *SOX9* mRNA expression in HT-115 CRC cells inducibly overexpressing GFP control, WT SOX9, mutant SOX9-228, mutant SOX9-303, or mutant SOX9-379 in the presence or absence of 0.5 *μ*g/mL doxycycline (DOX) using quantitative real time-PCR. Data expressed as mean ± SD of 3 technical replicates. (C) V5-tagged proteins and *β*-actin (loading control) protein expression in HT-115 CRC cells overexpressing V5-tagged WT and mutant SOX9 using 0.5 *μ*g/mL doxycycline by immunoblot. (D) Heatmap indicating V5-ChIP of HT-115 CRC cells inducibly overexpressing V5-tagged WT and mutant SOX9 in the presence of 0.5-*μ*g/mL doxycycline. (E) Heatmap showing H3K27Ac-ChIP of HT-115 CRC cells inducibly overexpressing V5-tagged WT and mutant SOX9 in the presence of 0.5-*μ*g/mL doxycycline. (F) Integration of RNA-seq and V5-ChIP data for WT and mutant SOX9 using BETA (Binding and Expression Target Analysis). DIM, dimerization domain; eGFP, enhanced green fluorescent protein; GFP, green fluorescent protein; HMG, DNA-binding high mobility group; mRNA, messenger RNA; PQA, PQA-rich domain.

**Table.** Genes Mutated in CRC in a Heterogenous Fashion Based on TCGA Analysis

A	B	C	D	E	F	G	H
Gene	Total oncogenic annotated mutations	Mutant samples with diploid or gain copy #	<50% Allele fraction mutations (oncogenic, exclude deletions)	Proportion heterozygous	Max allele fraction	% Samples with gene mutated (in data set)	Predicted function
TP53	328	78	48	0.14634	1	60%	Dominant negative
KRAS	219	197	144	**0.65753**	0.95	42%	GoF
PIK3CA	147	141	130	**0.88435**	0.86	28%	GoF
SMAD4	57	20	17	0.29825	0.89	16%	LoF
TCF7L2	40	29	24	**0.60000**	0.74	14%	Unknown biological effect/likely LoF
SOX9	54	46	41	**0.75926**	0.89	13%	Unknown biological effect/likely LoF
BRAF	56	55	49	**0.87500**	0.7	12%	GoF
ARID1A	46	41	38	**0.82609**	0.64	11%	Likely LoF
RNF43	38	36	33	**0.86842**	0.76	10%	Likely LoF
PTEN	46	37	34	**0.73913**	0.71	9%	LoF/unknown biological effect
AXIN2	22	20	20	**0.90909**	0.58	7%	Likely LoF
CTNNB1	18	16	10	**0.55556**	0.85	7%	GoF
ERBB2	9	9	5	**0.55556**	0.96	7%	GoF
NRAS	30	22	15	0.50000	0.86	7%	GoF
SMAD2	25	15	12	0.48000	0.81	7%	Likely LoF
MYC	4	4	4	**1.00000**	**0.47**	6%	Unknown biological effect
SMAD3	13	9	5	0.38462	0.88	6%	Likely LoF
RB1	7	7	7	**1.00000**	**0.39**	5%	Likely LoF
CDH1	8	7	7	**0.87500**	0.52	4%	Likely LoF
RBM10	8	7	3	0.37500	0.91	4%	Likely LoF
CDKN2A	2	2	2	**1.00000**	**0.38**	2.30%	Likely LoF
RHOA	4	4	4	**1.00000**	**0.38**	1.70%	Unknown/likely LoF/likely GoF

Gene	Total oncogenic annotated mutations	Mutant samples with diploid or gain copy #	<50% Allele fraction mutations (oncogenic, exclude deletions)	Proportion heterozygous	Max allele fraction	% Samples with gene mutated (in data set)
CSMD3	156	154	149	**0.95513**	0.68	23%
CSMD1	147	113	107	**0.72789**	0.91	23%
MUC6	38	37	35	**0.92105**	0.61	6%
FBXL7	33	31	26	**0.78788**	0.75	6%
PCBP1	25	25	24	**0.96000**	**0.5**	5%
TCF4	19	18	17	**0.89474**	**0.5**	5%
SOX11	14	12	11	**0.78571**	0.55	2.50%
TCF7	10	10	8	**0.80000**	0.55	2.50%
TGFB1	8	8	8	**1.00000**	**0.35**	1.50%

Mutations Dataset: Colorectal Adenocarcinoma (TCGA, PanCancer Atlas). Genes with a proportion of heterozygous alleles >0.5 are bolded in the proportion heterozygous column.

GoF, gain of function; LoF, loss of function; TCGA, The Cancer Genome Atlas.

## Abbreviations used in this paper:

CRC: colorectal cancer
H3K27Ac: H3 lysine 27 acetylation
WNT: wingless-related integration site
WT: wildtype

## References

[1] Liu Y, Cancer Cell 2018;33:721–735.e8. 29622466 10.1016/j.ccell.2018.03.010 PMC5966039

[2] Blache P, J Cell Biol 2004;166:37–47. 15240568 10.1083/jcb.200311021 PMC2172132

[3] Javier BM, Oncotarget 2016;7:50875–50882. 27248473 10.18632/oncotarget.9682 PMC5239443

[4] Vasaikar S, Cell 2019;177:1035–1049.e19. 31031003 10.1016/j.cell.2019.03.030 PMC6768830

[5] Liang X, Gastroenterology 2022;162:209–222. 34571027 10.1053/j.gastro.2021.09.044 PMC10035046

[6] Giacomelli AO, Nat Genet 2018;50:1381–1387. 30224644 10.1038/s41588-018-0204-y PMC6168352

[7] Boettcher S, Science 2019;365:599–604. 31395785 10.1126/science.aax3649 PMC7327437

[8] Sinha A, Sci Adv 2021;7:eabe0849.33597243

[9] Fumagalli A, Cell Stem Cell 2020;26:569–578.e7. 32169167 10.1016/j.stem.2020.02.008 PMC7118369

