# Supplementary material for: Truncating SOX9 Alterations Are Heterozygous Null Alleles in Genome-Stable Colorectal Cancer
Source: Gastro Hep Adv. 2022 May 2;1(5):709–13. doi: 10.1016/j.gastha.2022.04.011 (PMC10448869; doi:10.1016/j.gastha.2022.04.011)
Supplement: Supplemental Material [file mmc1.pdf]

SUPPLEMENTAL METHODS

Methods

Chromatin Immunoprecipitation followed by DNA-sequencing (ChIP-seq). HT115 cells were washed with PBS and crosslinked with 1% formaldehyde for 10 minutes for H3K27ac immunoprecipitation or crosslinked with two agents starting with 2 mM DSG (Pierce) for 45 minutes at room temperature followed by 1 mL of 1% formaldehyde for 10 minutes for the V5. Cross-linked cell lines were quenched with 0.125 M glycine for 5 minutes at room temperature. Cross-linked material was resuspended in 1% SDS (50 mM Tris-HCl pH8, 10 mM EDTA) and sonicated for 5 minutes with a Covaris E220 instrument (5% duty cycle, 140 Peak Incident Power, 200 Cycles per burst, 1 mL AFA Fiber milliTUBEs). Soluble chromatin (5 µg) was immunoprecipitated with 10 µg of H3K27ac (Diagenode C15410196 antibody) or 40 ug chromatin with 10ug V5 Tag Antibody (Invitrogen Cat# R96025 Lot# 1949337). ChIP-seq libraries were constructed using Accel-NGS 2S DNA library kit from Swift Biosciences. Fragments of the desired size were enriched using AMPure XP beads (Beckman Coulter). 36-bp paired-end reads were sequenced on a Nextseq instrument (Illumina).

ChIP-seq analysis. The ChiLin pipeline 2.0.0[1] was used for quality control and pre-processing of the data. We used Burrows-Wheeler Aligner (BWA Version: 0.7.17-r1188) as a read mapping tool, and Model-based Analysis of ChIP-Seq (MACS2)[2] (v2.1.0.20140616) as a peak caller using default parameters. CEAS analysis is used to annotate resulting peaks with genome features. Differential analysis of peaks was determined by DESeq (Anders S et al). BETA[3] was used to integrate ChIP-seq of transcription factors or chromatin regulators with differential gene expression data to infer direct target genes. Super-enhancers were called by ROSE[4] in H3K27ac ChIP-seq data. Cistrome toolkit was used to probe which factors might regulate the user-defined genes. Genomic Regions Enrichment of Annotations Tool (GREAT)[5] was used to annotate peaks with their biological functions. Conservation plots were obtained with the Conservation Plot (version 1.0.0) tool available in Cistrome.

ChIP-seq data visualization. Normalized profiles corresponding to read coverage per 1 million reads were used for heatmaps and for visualization using the integrative genomics viewer (IGV)[6]. Wiggle tracks were visualized using the integrative genomics viewer. Heat maps were prepared using deepTools (version 2.5.4)[7]

Cell Culture, Lentivirus Packing, and Transduction. All cell lines were maintained at 37 °C with 5% CO2. The human colorectal cancer cell lines were obtained from the CCLE core facility and used at early passage for the experiments. HEK293T, HT-115, HT-29, and COLO-205 cells were maintained in DMEM medium supplemented with 10% FBS and 1% penicillin/streptomycin. LS513,

LS123, SW1463, and LS180 cells were cultured in RPMI 1640 containing 10% FBS and 1% penicillin/streptomycin. CL-40 cells were cultured in DMEM/F12 (1:1) supplemented with 20% FBS and 1% penicillin/streptomycin.

To generate lentiviruses, expression vectors were co-transfected into HEK293T cells with the lentiviral packaging constructs psPAX2 and pMD2.G (VSV-G) in a 1:1:1 ratio using X-tremeGENE 9 DNA Transfection Reagent (Roche) according to the manufacturer's instructions. Cell culture media was changed the following day and lentiviral supernatant was harvested 48 h and 72 h later and filtered through a 0.45 μm filter (Millipore). Lentiviruses were aliquoted and stored at - 80 °C until use.

To perform lentiviral infection, the CRC cells were plated in a 6-cm dishes and infected with 0.5-1 mL virus in media containing 8 mg/mL polybrene overnight.

Generation of stable cell lines. All genetically manipulated colon organoid lines were generated using the protocol described here[8]. To generate V5-tagged inducible expression of SOX9 and truncated variants, PLIX403 vectors were used and 15 μg/ml blasticidin selection was started 24 hours after infection.

RNA isolation and qPCR.
Total RNA was isolated using the RNeasy Mini Kit (Qiagen, Germantown, MD, USA) and cDNA was synthesized using the iScript™ Reverse Transcription Supermix for RT-qPCR (Bio-Rad) according to the manufacturer's instructions. Gene-specific primers for SYBR Green real-time PCR were either obtained from previously published sequences or designed by PrimerBLAST (https://www.ncbi.nlm.nih.gov/tools/primer-blast/) and synthesized by Integrated DNA Technologies or ETON biosciences. Real-time PCR was performed and analyzed using CFX96 Real-Time PCR Detection System (Bio-Rad Laboratories, Inc., Hercules, CA) and using Power SYBR Green PCR Master Mix (Thermo Fisher Scientific) according to the manufacturer's instructions. Relative mRNA expression was determined by normalizing to *GAPDH* expression, which served as an internal control.

Immunoblot and antibodies. Immunoblot analysis was performed as previously described[8]. Briefly, cells were lysed in RIPA buffer supplemented with a protease inhibitor cocktail (Roche). Whole cell extracts were resolved by SDS-PAGE, transferred to PVDF membranes, and probed with indicated primary antibodies. Bound antibodies were detected with horseradish peroxidase (HRP)-conjugated secondary antibodies and chemiluminescent HRP substrate.

The following primary antibodies were used for western blotting (all from Cell Signaling Technologies, Beverly, MA, USA, unless otherwise indicated): anti-SOX9 (#82630, 1:1,000), anti-β-Actin (A5441, 1:1,000, Sigma), and anti-V5 (R960-25, 1:2,500, Thermo Fisher)

Allele frequency of the somatic mutations. To determine the allele frequency of the *SOX9* somatic mutation, we queried the genomic information from colorectal adenocarcinoma patient samples included in the TCGA PanCan Atlas dataset. When the mutant allele frequency of a somatic mutation is less than or equal to 0.5, the mutation has been called as heterozygous mutation. The results are based upon data generated by TCGA and made available through cBioPortal[9].

For the broader analysis summarized in Table 1, we performed the following steps: An initial gene list was composed using the combination of the top 20 genes mutated in CRC according to the TCGA database and most commonly mutated genes in CRC as found by Liu et al in a comprehensive genomic analysis of gastrointestinal cancers[1]. This resulted in an initial list of 50 genes, which were narrowed down to 30 genes of interest due to having known oncogenic or tumor suppressive functions. These 30 genes were queried in cBioPortal using the CRC dataset containing 526 CRC patient samples titled “Colorectal Adenocarcinoma (TCGA, PanCancer Atlas)”. For each gene queried, total number of oncogenic or likely oncogenic mutations according to cBioPortal annotations were recorded in column B. The number of mutations with copy number recorded as “diploid” or “gain” were totaled in column C, and of these remaining oncogenic mutations the number with an allele fraction less than 50% was totaled and recorded in column D. The proportion heterozygous in column E was calculated by dividing column D by column B; the percentages at or below 50% have been bolded. The maximum allele fraction, percentage of total samples with a specific gene mutation, and the predicted mutation functions as annotated by cBioPortal are listed in columns F, G, and H respectively. Maximum allele fractions of 0.5 or less have been bolded. Several mutations on the original list of interest had no annotated mutations in cBioPortal, and are provided at the bottom of the table in a separate section.

Statistical Analysis and reproducibility. Data are represented as mean ± s.d unless indicated otherwise. For each experiment, either independent biological experiments or technical replicates are as noted in the figure legends. Statistical analysis was performed using Microsoft Office statistical tools or in Prism 7.0 (GraphPad). Pairwise comparisons between groups (that is, experimental versus control) were performed using an unpaired two-tailed Student’s *t*-test or Kruskal–Wallis test as appropriate unless otherwise indicated.

ACKNOWLEDGEMENTS

We thank Yingtian Xie, Paloma Cejas, Klothilda Lim, and Henry W. Long of the Center for Functional Cancer Epigenetics (CFCE) for technical assistance on ChIP-seq experiments; Ramesh Shivdasani, Manav Korpal, Adam Sperling, Shridar Ganesan, Mathew Hemming, David Liu, Doug Micalizzi, Harshabad Singh and Ankur Nagaraja for insightful discussions; Dana-Farber/Harvard Cancer Center for the use of the Specialized Histopathology Core, which provided histology and immunohistochemistry service; Harvard Digestive Disease Center and NIH grant P30DK034854 for core services, resources, technology, and expertise. Dana-Farber/Harvard Cancer Center is supported in part by an NCI Cancer Center Support Grant # NIH 5 P30 CA06516. This work was funded by the Perry Fellowship, Claudia Barr Award, and Virtual Scholar Award from the Department of Defense (CA201084) to N.S.S.

SUPPLEMENTARY FIGURES

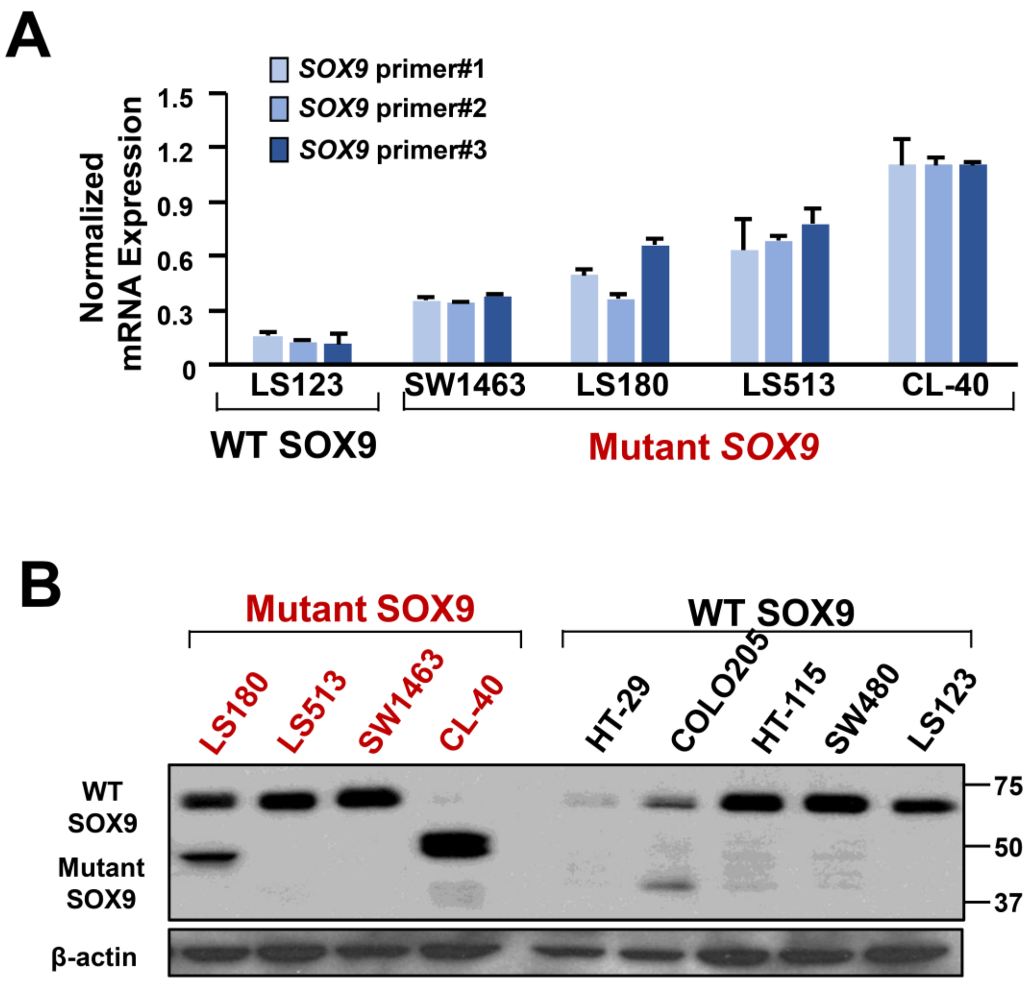

Supplementary Figure 1. *SOX9* mutations lead to expression of truncated variants
(A) qRT-PCR analysis of *SOX9* expression in five CRC cell lines. Cell lines are indicated as containing either wildtype or mutated *SOX9*. Data expressed as mean ± S.D.
(B) Immunoblot showing SOX9 and β-actin (loading control) expression in nine CRC cell lines containing wildtype and mutated *SOX9*.

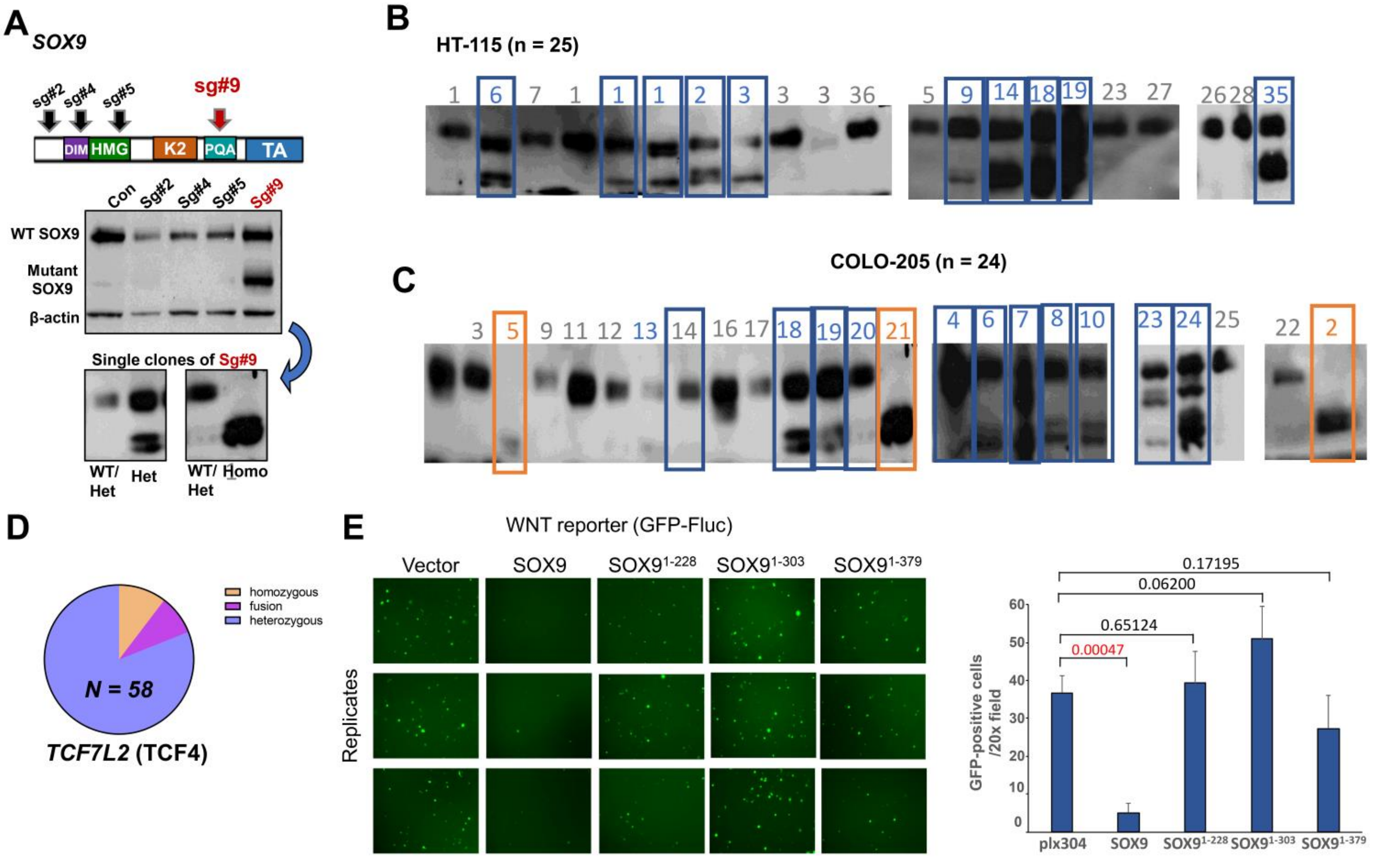

Supplementary Figure 2. CRISPR/Cas9-induced *SOX9* mutations are preferentially heterozygous

(A) Schematic showing Cas9 recruitment to specific locations along *SOX9* gene body by indicated sgRNAs. SOX9 and β-actin (loading control) protein expression in COLO-205 CRC cell line stably expressing control or indicated sgRNA against SOX9 by immunoblot.

(B) Immunoblots showing SOX9 expression in HT-115 single cell clones stably expressing sgRNA#9 which directs Cas9 to the c-terminus of SOX9.

(C) Immunoblots showing SOX9 expression in COLO205 single cell clones stably expressing sgRNA#9 which directs Cas9 to the c-terminus of SOX9..

(D) Analysis of alterations in the WNT pathway effector encoded by *TCF7L2* in CRC cell lines in the CCLE database. Chart representing distribution of homozygous and heterozygous mutations as well as fusions as determined by Cbioportal and allele fraction analysis (please see methods).

(E) Immunofloursent images (left) and quantification (right) of WNT-GFP-reporter CRC cell line expressing vector control, SOX9, or three truncated SOX9 mutants

SUPPLEMENTAL REFERENCES

1. Qin Q, Mei S, Wu Q, et al. ChiLin: a comprehensive ChIP-seq and DNase-seq quality control and analysis pipeline. BMC Bioinformatics 2016;17:404.
2. Zhang Y, Liu T, Meyer CA, et al. Model-based analysis of ChIP-Seq (MACS). Genome Biol 2008;9:R137.
3. Wang S, Sun H, Ma J, et al. Target analysis by integration of transcriptome and ChIP-seq data with BETA. Nat Protoc 2013;8:2502-15.
4. Whyte WA, Orlando DA, Hnisz D, et al. Master transcription factors and mediator establish super-enhancers at key cell identity genes. Cell 2013;153:307-19.
5. McLean CY, Bristor D, Hiller M, et al. GREAT improves functional interpretation of cis-regulatory regions. Nat Biotechnol 2010;28:495-501.
6. Thorvaldsdottir H, Robinson JT, Mesirov JP. Integrative Genomics Viewer (IGV): high-performance genomics data visualization and exploration. Brief Bioinform 2013;14:178-92.
7. Ramirez F, Dundar F, Diehl S, et al. deepTools: a flexible platform for exploring deep-sequencing data. Nucleic Acids Res 2014;42:W187-91.
8. Liang X, Duronio GN, Yang Y, et al. An Enhancer-Driven Stem Cell-Like Program Mediated by SOX9 Blocks Intestinal Differentiation in Colorectal Cancer. Gastroenterology 2022;162:209-222.
9. Cerami E, Gao J, Dogrusoz U, et al. The cBio cancer genomics portal: an open platform for exploring multidimensional cancer genomics data. Cancer Discov 2012;2:401-4.
